# Monitoring of Temperature Fatigue Failure Mechanism for Polyvinyl Alcohol Fiber Concrete Using Acoustic Emission Sensors

**DOI:** 10.3390/s120709502

**Published:** 2012-07-11

**Authors:** Dongsheng Li, Hai Cao

**Affiliations:** School of Civil Engineering, Dalian University of Technology, Dalian 116024, China; E-Mail: dlutcaohai@126.com

**Keywords:** PVA fiber-reinforced concrete, temperature fatigue, acoustic emission, damage assessment, health monitoring

## Abstract

The applicability of acoustic emission (AE) techniques to monitor the mechanism of evolution of polyvinyl alcohol (PVA) fiber concrete damage under temperature fatigue loading is investigated. Using the temperature fatigue test, real-time AE monitoring data of PVA fiber concrete is achieved. Based on the AE signal characteristics of the whole test process and comparison of AE signals of PVA fiber concretes with different fiber contents, the damage evolution process of PVA fiber concrete is analyzed. Finally, a qualitative evaluation of the damage degree is obtained using the kurtosis index and *b*-value of AE characteristic parameters. The results obtained using both methods are discussed.

## 1. Introduction

The application of fiber-reinforced concrete in various structures is increasing. Fiber can improve the tensile strength of concrete, its resistance to deformation, durability, dynamic effects, and so on [1]. The crack resistance and toughness of fiber are better than those of plain concrete because an increase in fiber content enhances fiber resistance and delays cracking of the material. The winter season in north China, the alternation of the four seasons, the day and night temperature differences, among other factors, expose concrete structures during their service life to temperature freeze-thaw conditions, which cause temperature fatigue damage. Polyvinyl alcohol (PVA) fiber is an environmentally friendly, reinforced cement material ideal for use in northern China because of its good dispersion characteristics, ease of use in construction, good affinity with cement, alkali resistance, and resistance to the effects of climate and weather conditions. PVA can improve the antifreeze and anti-fatigue properties of concrete. Thus, the durability and frost resistance of concrete materials can be improved when mixed with PVA fiber.

Acoustic emission (AE), a real-time monitoring technology, has many applications in material damage identification [2]. The AE technique is used for monitoring the evolution of damage in substandard concrete materials [3,4] and to monitor matrix cracking and failure of different interfaces, especially in fiber concrete [5,6]. Previous studies showed that the AE technique can detect and possibly identify damage mechanisms in fiber-reinforced concrete by analyzing AE parameters. Among the parameters, AE energy, amplitude, and duration are usually used to examine the damage process zone [7–9]. Sometimes, however, the commonly examined AE parameters alone cannot correctly explain the mechanism of material failure. Several AE waveforms and spectral parameters were investigated to harness the full potential of the AE technique in studying the deformation of materials under stress [10]. However, both direct AE parameters and waveform only qualitatively describe the damage process. Quantitative methods attempt to describe the nature of a source, for example, using moment tensor inversion techniques [11–13]. However, quantitative methods cannot be easily applied in large structures, so qualitative methods are mostly used in the evaluation of material damage. Based on AE characteristic parameters, some indirect combination parameters are proposed to quantify the damage level of RC structures. Common AE qualitative methods include the *b*-value', Felicity ratio, historical index versus severity index, relaxation ratio, kurtosis index, and RA value [14–19]. In RC structures, the *b*-value theory and Felicity ratio are the most basic methods of analysis. The *b*-value is defined as the log-linear slope of the frequency-magnitude distribution of acoustic emissions, and shows good agreement with the process of fracture development in concrete. The Felicity ratio that has been developed into a standard can be used to determine if major structural defects are present in the material.

In this study, the damage of temperature fatigue load to PVA fiber concrete specimens is monitored using AE technology. The influence of different PVA fiber contents in the freezing-thawing resistance durability of concrete material is also investigated. The damage evolution process of specimens is studied using the correlation analysis method for AE parameters.

## 2. Experiments

### 2.1. Test Specimens

According to “Standard test methods of long term performance and durability of ordinary concrete (GB/T 50082-2009)”, the specimen had a length of 400 mm and a cross-section of 100 mm × 100 mm. The mixture ratio is presented in Table 1 and the PVA fiber characteristics are shown in Table 2. The experiment utilized high-quality fine aggregate sand with a fineness modulus of 2.13 and 1.5% silt content. The II district-level was qualified. Standard curing lasted for 28 days. The three-point bending experiments for the determination of the specimen toughness were performed. The testing results were listed in Table 3. The results were shown that the increase of fiber content improved the material's ultimate load and toughness.

### 2.2. Temperature Fatigue Test

**Figure 1.**
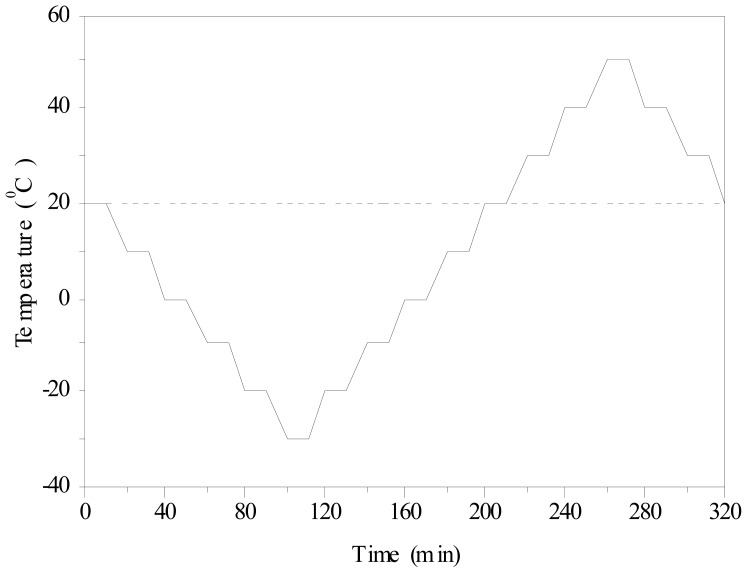
Temperature loading procedures.

The temperature fatigue test protocol was also referred to the standard for GB/T 50082-2009 in China. Before temperature fatigue testing, the specimens from each mixture ratio group were taken and soaked in water for 4 days. Subsequently, the soaked specimen were put into the rapid freeze-thaw testing machine to simulate hot and wet testing cases for the temperature fatigue test. Humidity was above 90% in the control group when the environment was more than 25 °C. The temperature ranged from 50 °C to −30 °C, changing by 10 °C for every step. Heating or cooling at 10 °C lasted 10 min. Thus, temperature was kept constant for 10 min for 320 min a cycle for 50 cycles, as shown in Figure 1.

### 2.3. AE Acquisition System

**Figure 2.**
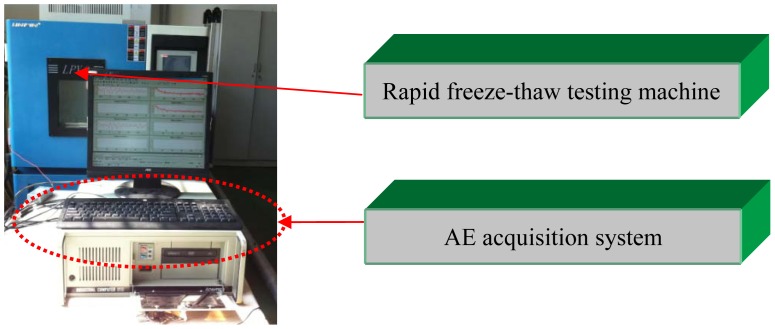
PVA concrete temperature fatigue damage testing experimental device.

**Figure 3.**
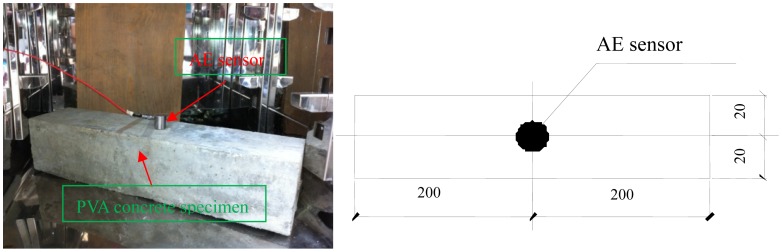
AE sensors arrangement (units: mm).

AE signals were collected by an AE acquisition system from the American Physical Acoustics Corporation. The sensor type is R15-ALPHA. Its resonant frequency is 150 KHz. Acquisition system parameters are as follows: the pre-amplifier gain for 20 dB, the main amplifier for 30 dB, threshold for 46 dB and sample rate for 5 MHz. One AE sensor was installed in the concrete specimen to assess the integrality of the structure. The AE sensor was fixed using tape. Schematic diagram of AE sensor arrangement is in Figures 2 and 3.

## 3. Results and Discussion

### 3.1. PVA Fiber Concrete Temperature Fatigue Damage AE Characteristic Parameters

**Figure 4.**
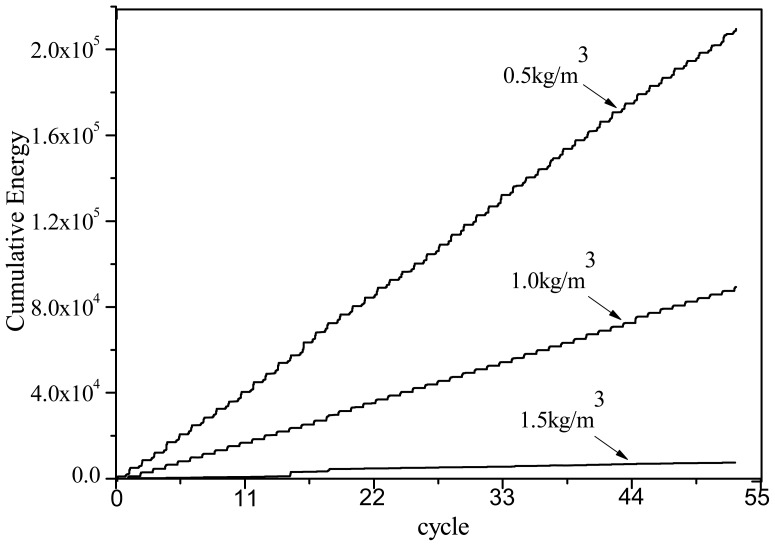
AE cumulative energy *vs.* time.

**Figure 5.**
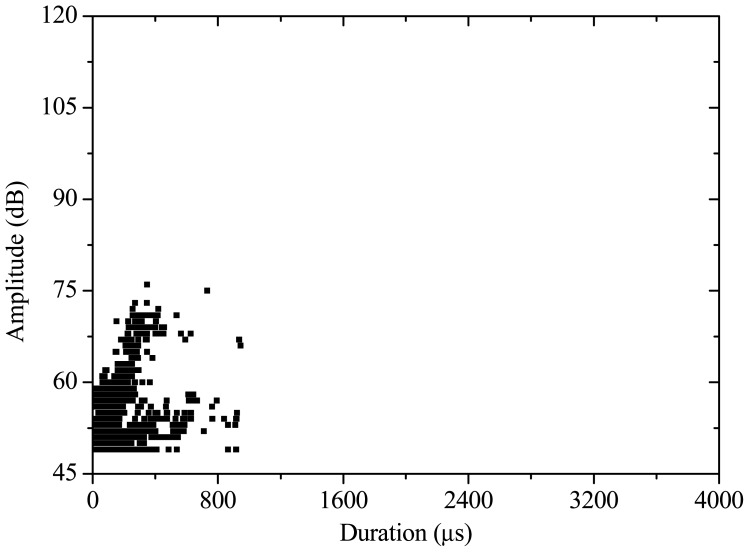
AE amplitude *vs.* duration (1.5 kg/m^3^).

**Figure 6.**
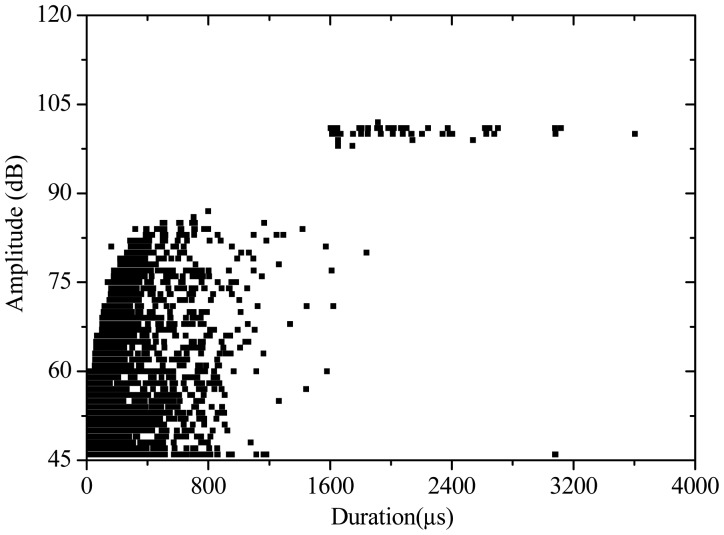
AE amplitude *vs.* duration (1.0 kg/m^3^).

**Figure 7.**
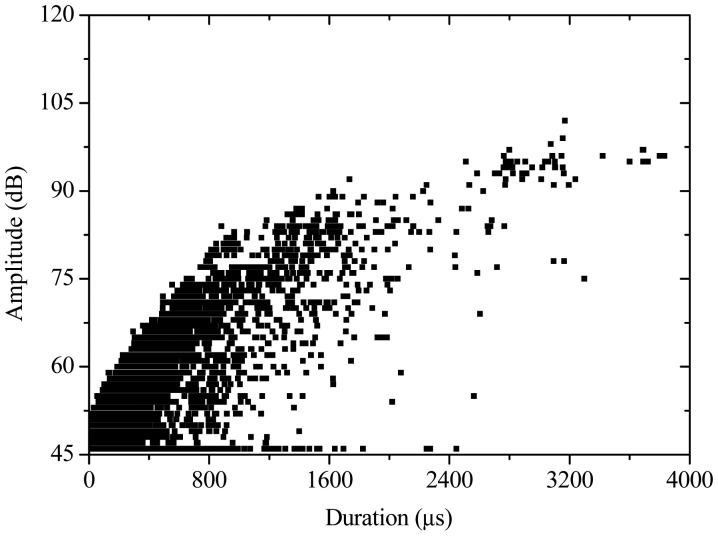
AE amplitude *vs.* duration (0.5 kg/m^3^).

The AE characteristic parameters of PVA fiber concrete specimen temperature fatigue damage are shown in Figures 4–7. In contrast to the AE signal figure, all specimen AE signals slowly increase. According to Figure 4, the AE signal energy of the different specimens is reduced with the increase of fiber contents. This is reasonable because the deformation resistance capacity for high fiber content material is better than with low content fiber. This can effectively prevent inner crack formation in the specimens under temperature fatigue load.

Moreover, based on the relationship of AE amplitude *vs.* duration (Figures 5–7), the AE signal varies with different PVA fiber concrete contents. For the specimen with fiber content of 1.5 kg/m^3^, AE amplitude is lower than 75 dB, and the duration of the signal is less than 1,000 μs. The PVA fiber exhibits a strong contribution in resisting crack propagation. Thus the specimen damage is small. For the specimen with fiber content of 1.0 kg/m^3^, AE amplitude is lower than 85 dB, and the duration is less than 1,600 μs. However, some high-amplitude and long-duration AE signals are observed when the AE amplitude reaches 100 dB. The reason is because the decreased fiber content leads to the crack propagation resistance ability becoming weaker. Accordingly, when the fiber content is reduced to 0.5 kg/m^3^, the distribution of AE amplitude *vs.* duration is more scattered. Thus, AE amplitude is higher and duration is longer. Lower PVA fiber content in the concrete specimen also reduces the antifreeze ability. Many cracks appear on the specimen, the damage condition is more complex, and damage is more pronounced.

**Figure 8.**
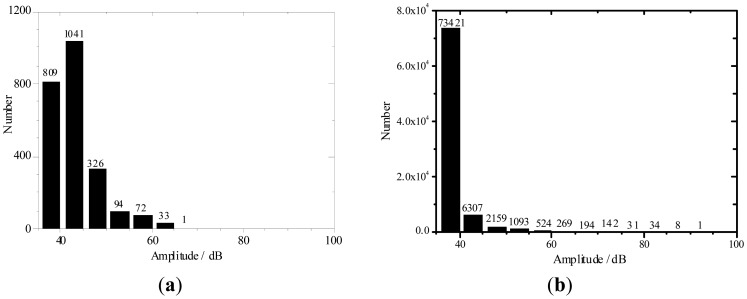

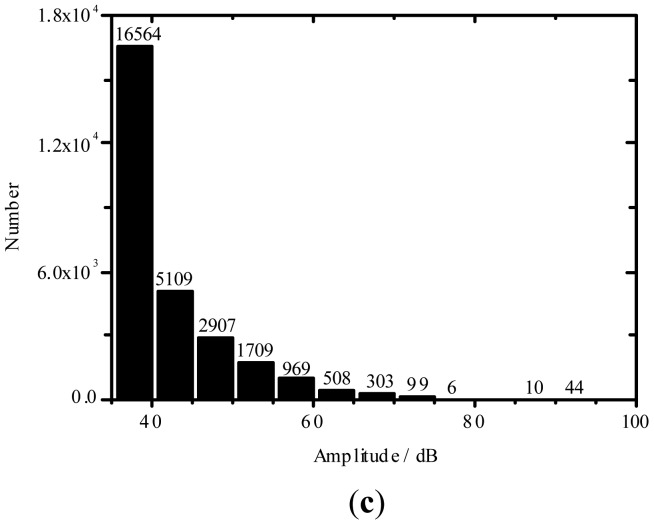
AE amplitude statistical distributions for PVA fiber contents concrete with: (**a**) 1.5 kg/m^3^, (**b**) 1.0 kg/m^3^ and (**c**) 0.5 kg/m^3^.

AE amplitude statistical distributions for different volumetric PVA fiber content concrete were listed in Figure 8. Among the three different fiber content concretes, the 0.5 kg/m^3^ fiber content specimens' AE activity number was maximum. The high amplitude AE events were increasing with decreasing fiber contents. This is reasonable because each fiber pull-out event is a potential AE hit and the pull-out events increase with the fiber volume content [5]. The testing results verified that the high PVA fiber contents concrete could improve the concrete antifreeze ability and concrete damage degree was reduced.

### 3.2. PVA Fiber Concrete Temperature Fatigue Damage Evolution Describes Based on AE Amplitude over Time

**Figure 9.**
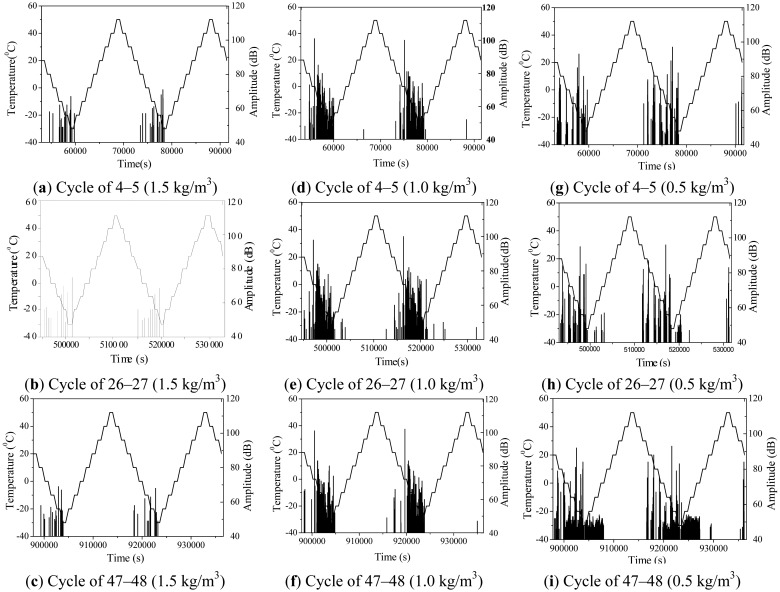
Temperature fatigue load and AE amplitude *vs.* time.

The AE signal amplitude of different fatigue load phases is shown in Figure 9. In Figure 9(a–c), less AE signal is observed for the specimen with 1.5 kg/m^3^ fiber content during the whole cycle. The amplitude is low and rarely any AE signal exists in temperature increase stage. The specimens show little damage. In Figure 9(d–f), more AE signal is noted for the specimen with 1.0 kg/m^3^ fiber content during the whole cycle. Amplitude is also higher. When the temperature goes down to 0 °C, the amplitude reaches the maximum value, and the specimen damage is bigger, but the damage process is still weak. In Figure 9(g–i), abundant AE signal and high amplitude are observed for the specimen with 0.5 kg/m^3^ fiber content in the first several cycles. As the fatigue test is performed, the AE signal increases with reduction in fiber content of the specimen. When the temperature is below 0 °C, a large AE signal occurs. Large, high-amplitude signals show more serious damage in the specimen, especially in the final several cycles where the specimen antifreeze ability is significantly reduced.

The antifreeze property of the material with the lowest fiber content is substantially reduced because it contains free water. When the temperature is below freezing point, internal temperature stress in the material is caused by its volume expansion, and repeated freeze-thaw causes serious damage to the specimen. Moreover, PVA fiber acts as an air-entraining agent. When concrete is reinforced with PVA fiber, the internal air content increases and gas porosity increases, which can delay the initiation and propagation of the micro cracks in the matrix material. When the temperature continues to decrease below the freezing point, free water becomes ice and cracks begins to form and expand by frozen-heave stress in the material. PVA fibers bear more and more of the frozen-heave stress with micro crack formation and extension, and reduce matrix micro cracks of concrete and resist the matrix material damage.

When the rising temperature, the frozen-heave stress decreases gradually and material damage will not increase further, as shown in Figure 9(a–i). When the fiber content is small and the fiber tensile ability is reduced by repeated frozen-heave stresses, fiber fatigue damage occurs and this gradually leads to broken wire damage. With broken wire damage, the cement matrix damage and degradation rate increase, as shown in Figure 9(g–i). Therefore, PVA fiber-reinforced concrete material can show improved frost resistance ability, and a fiber content of 1.5 kg/m^3^ in the materials exhibits the best frost resistance.

## 4. Damage Evaluation for PVA Fiber Concrete Based on the AE Data

### 4.1. Evaluation PVA Fiber Concrete Damage Evolution by Kurtosis Index

The instantaneous index is often adopted to evaluate possible defects in rotating machinery and machinery service ability in vibration analysis. Kurtosis index is a measure of the impulsive nature of the signal. Thus it can be used to evaluate potential defects and its service capabilities for PVA fiber concrete. The kurtosis index was used to analyze AE signal in the present study. It is calculated as follows [18]:

(1) $$\text{Kurtosis}=\frac{\frac{1}{N}\overset{N}{\underset{n=1}{\text{∑}}}{\left(x(n)-\frac{1}{N}\overset{N}{\underset{n=1}{\text{∑}}}x(n)\right)}^{4}}{{\left[\frac{1}{N}\overset{N}{\underset{n=1}{\text{∑}}}{\left(x(n)-\frac{1}{N}\overset{N}{\underset{n=1}{\text{∑}}}x(n)\right)}^{2}\right]}^{2}}$$

where *N* is the length of each group AE characteristic parameter (energy) *x(n)*.

Kurtosis indexes can show the differences between different damage stages. When the kurtosis value is smaller; the fatigue damage is the smaller, and the greater the kurtosis value, the greater the fatigue damage is [18]. In structural fatigue damage monitoring by AE, AE signals of each load cycle are as a group to calculate kurtosis value.

**Figure 10.**
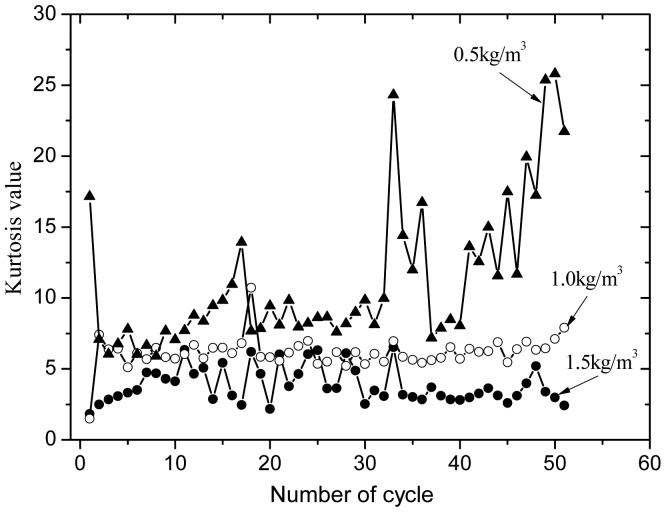
Kurtosis index for PVA fiber concrete.

The kurtosis index is shown in Figure 10. It is reduced in general when fiber contents increase. On the 16th and 32nd cycle load sets, the kurtosis indexes of different fiber contents appear as extreme values, which suggests that the internal damage is serious. The analysis reveals that in temperature fatigue load, the internal part of the concrete produces temperature stress, and the PVA fiber bears part of the stress. With increasing damage, this internal strain is continuously accumulated. When the strain reaches a certain limit, the accumulated energy is released and the temperature stress inside the specimen is reduced. In later temperature fatigue load (after 40 cycles), the kurtosis index of the 0.5 kg/m^3^ fiber content specimens increases rapidly, which results in increased and serious specimen damage. In this case, the function of PVA fiber in resisting stress and deformation weakens. Calculation of the kurtosis index reveals that antifreeze ability is more ideal for specimens with fiber content of 5 kg/m^3^.

### 4.2. Evaluation PVA Fiber Concrete Damage Evolution by b-Value

The *b*-value is an effective method in studying the fracture mechanism. The *b*-values for different materials also show different ranges of values and reflect the degree of damage degradation for the concrete material. Schumacher *et al.* [20] proposed that if there are several low amplitude values for an AE event and the signal is more dispersed with b > 1.0, microscopic cracks will form. In this experiment, each cycle of temperature load is performed for a data group, and then *b*-value is calculated. The *b*-value is calculated in the AE technique as follows [14]:

(2) $$\log_{10}N=a-b\times A_{dB}$$

where *N* is the AE event, A_dB_ is the peak amplitude of the AE event in decibels, and *a* and *b* are empirical constants. The *b*-value is obtained by the least square method.

**Figure 11.**
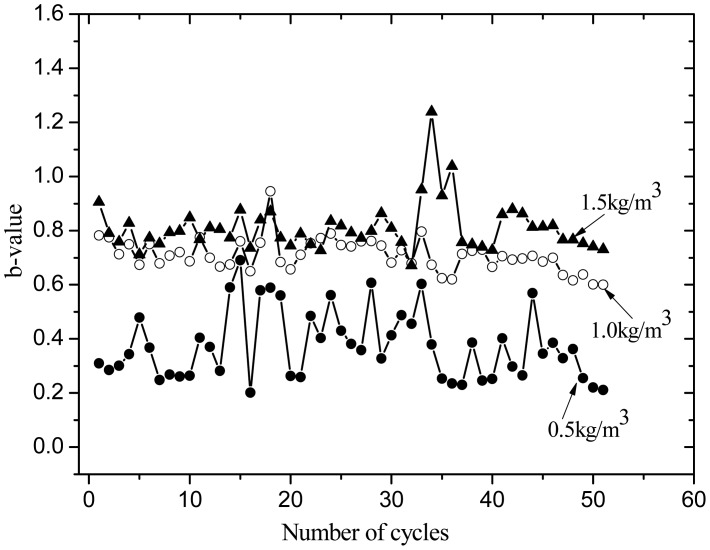
*b*-value for PVA fiber concrete.

The *b*-value is shown in Figure 11. When the fiber content is greater, the *b*-value is higher. A low fiber content has a lower *b*-value for higher amplitude events (as shown in Figures 8) from potential macro-cracking. At the initial stage, the fibers bear part of the stress, which generates very small micro cracks and expansions. On the 16th cycle load set, the *b*-value becomes small. This indicates that internal micro cracks appear in the specimens, showing a redistribution of internal stress. With increasing temperature fatigue cycles, the bearing stress in the PVA fiber increases. The damage is slightly different from that at the initial stage. The temperature fatigue resistance performance does not significantly change and brittle failure is not expected to occur. The fatigue cycle last stage is the damage acceleration stage, especially for specimens with fiber content of 0.5 kg/m^3^. The *b*-value is reduced and reaches a minimum value. Through visual inspection, some cracks appeared on the fiber concrete surface. The inner micro cracks had developed external macro cracks. The specimens deteriorate rapidly. Damage in specimens with fiber content of 1.0 and 1.5 kg/m^3^ is still very small. The load and AE amplitude signals in Figures 4–7 reflect the laws. Thus, PVA fibers can effectively delay the deformation process in concrete material damage. When the fiber volume is high, the damage is small.

## 5. Conclusions

In this study, PVA fiber concrete temperature fatigue tests were used to obtain the AE characteristics parameters. An analysis of the degradation and damage evolution process was then performed. The main conclusions drawn from the experiment are as follows:

1. PVA fiber has a significant effect on the improvement of frost resistance in concrete, and effectively improves its durability. The higher the fiber content, the smaller the degree of damage.
2. The AE signals show high correlation with the damage of the PVA fiber concrete. The damage evolution process of PVA fiber concrete can be accurately expressed using the AE technique. Temperature fatigue load has a large influence on PVA fiber concrete damage and AE characteristic parameters.
3. The kurtosis index and *b*-value index can qualitatively evaluate damage status and the damage evolution process for PVA fiber concrete. As damage develops on the PVA fiber concrete, the kurtosis index increases and the *b*-value becomes small.

## Figures and Tables

**Table 1.** Concrete mixture ratio.

**P·O 42.5 Cement (kg/m^3^)**	**Sand (kg/m^3^)**	**Stone (kg/m^3^)**	**Water (kg/m^3^)**	**PVA fiber (kg/m^3^)**
432	587	1,190	200	0.5
432	587	1,190	200	1.0
432	587	1,190	200	1.5

**Table 2.** Fiber characteristics.

**Diameter**	**Tensile strength**	**Young's modulus**	**Breaking elongation**	**Length**
15 μm + 3	1,200 MPa	35 GPa	6–11%	6 mm	

**Table 3.** Mechanical properties for different fiber contents under the three-point bending test.

**PVA fiber content (%)**	**Maximum load (KN)**	**Flexural toughness (J)**
0	12.0	-
0.5	12.1	10.5
1.0	12.8	13.2
1.5	13.2	14.6
